# Factors Associated with Response to Systemic Corticosteroids in Active Ulcerative Colitis: Results from a Prospective, Multicenter Trial

**DOI:** 10.3390/jcm12144853

**Published:** 2023-07-24

**Authors:** Andreas Blesl, Andrea Borenich, Hans Peter Gröchenig, Gottfried Novacek, Christian Primas, Walter Reinisch, Maximilian Kutschera, Constanze Illiasch, Barbara Hennlich, Pius Steiner, Robert Koch, Wolfgang Tillinger, Thomas Haas, Gerhard Reicht, Andreas Mayer, Othmar Ludwiczek, Wolfgang Miehsler, Karin Steidl, Lukas Binder, Franziska Baumann-Durchschein, Stefan Fürst, Simon Reider, Christina Watschinger, Heimo Wenzl, Alexander Moschen, Andrea Berghold, Christoph Högenauer

**Affiliations:** 1 Department of Internal Medicine, Division of Gastroenterology and Hepatology, Medical University of Graz, 8036 Graz, Austria; 2 Institute for Medical Informatics, Statistics and Documentation, Medical University of Graz, 8036 Graz, Austria; 3 Brothers of Saint John of God Hospital, 9300 St. Veit an der Glan, Austria; 4 Department of Internal Medicine III, Division of Gastroenterology and Hepatology, Medical University of Vienna, 1090 Vienna, Austria; 5 Hospital Landstraße, 1030 Vienna, Austria; constanze.illiasch@wienkav.at (C.I.);; 6 Hospital Wels-Grieskirchen, 4600 Wels, Austria; 7 Department of Internal Medicine I, Gastroenterology, Hepatology, Endocrinology & Metabolism, Medical University of Innsbruck, 6020 Innsbruck, Austria; 8 Franziskus Hospital, 1050 Vienna, Austria; 9 Darmpraxis Salzburg, 5020 Salzburg, Austria; 10 Brothers of Saint John of God Hospital, 8020 Graz, Austria; gerhard.reicht@bbgraz.at; 11 University Hospital St. Pölten, 3100 St. Pölten, Austria; 12 Hospital Hall, 6060 Hall, Austria; 13 Brothers of Saint John of God Hospital, 5010 Salzburg, Austria; 14 Department of Internal Medicine II (Gastroenterology and Hepatology), Faculty of Medicine, Kepler University Hospital, Johannes Kepler University, 4021 Linz, Austria; 15 Christian Doppler Laboratory for Mucosal Immunology, Johannes Kepler University, 4021 Linz, Austria

**Keywords:** ulcerative colitis, systemic corticosteroids, inflammatory bowel disease

## Abstract

Background: Among patients with ulcerative colitis, 30–50% receive corticosteroids within the first five years after diagnosis. We aimed to reconsider their effectiveness in the context of the biologic era. Methods: In this prospective, multicenter study, patients with active ulcerative colitis (Lichtiger score ≥ 4) were eligible if initiating systemic corticosteroids. The primary endpoint was clinical response (decrease in the Lichtiger score of ≥50%) at week 4. Secondary endpoints included combined response defined as clinical response and any reduction in elevated biomarkers (CRP and/or calprotectin). Steroid dependence was assessed after three months. Results: A total of 103 patients were included. Clinical response was achieved by 73% of patients, and combined response by 68%. A total of 15% of patients were steroid-dependent. Activity of colitis did not influence short-term response to treatment but increased the risk for steroid dependence. Biologic-naïve patients responded better than biologic-experienced patients. Past smoking history (OR 5.38 [1.71, 20.1], *p* = 0.003), hemoglobin levels (OR 0.76 [0.57, 0.99] for higher levels, *p* = 0.045), and biologic experience (OR 3.30 [1.08, 10.6], *p* = 0.036) were independently associated with nonresponse. Conclusion: Disease activity was not associated with short-term response to systemic corticosteroids but was associated with steroid dependence in patients with active ulcerative colitis. Exposure to biologics negatively affects response rates.

## 1. Introduction

Ulcerative colitis (UC) is a chronic immune-mediated disease affecting the colonic mucosa [1]. Until now, systemic corticosteroids are recommended as induction therapy for moderate to severely active disease [2,3]. Still, the likelihood of a UC patient receiving corticosteroids within the first five years after diagnosis is 30–50% [4,5]. In historical cohorts from the 1960s with limited sample size, remission rates of around two-thirds to three-quarters were reported [6,7,8], but new data on efficacy and factors influencing and predicting response are rare. These newer studies have mainly focused on acute severe colitis and factors influencing the necessity of a rescue therapy or colectomy [9,10,11,12], while investigations on patients with moderately active UC are lacking.

Here we present the clinical results of an investigator-initiated, prospective, multicenter trial originally performed to evaluate predictive microbial alterations before initiation of systemic corticosteroids in patients with active UC [13]. We assessed clinical and biochemical response and aimed to determine demographic, disease-specific, and clinical factors influencing and predicting response to treatment.

## 2. Methods

### 2.1. Study Population

From May 2018 to December 2020, patients with active UC were recruited from 18 study centers in Austria. Patients were eligible if they were suffering from active disease and were scheduled for treatment with oral or intravenous systemic corticosteroids. The diagnosis of UC needed to be either established before study inclusion or confirmed later in patients with suspected UC at inclusion. The diagnosis was made according to the local practice. Active UC was defined as Lichtiger score ≥ 4 and severely active colitis as Lichtiger score > 10 [14,15]. Exclusion criteria included bacterial, viral, or parasitic infections and a diagnosis of Crohn’s disease. Stable ongoing medications could be continued during the study period. Corticosteroid dosing and tapering were performed in accordance with the clinical practice at each study center.

### 2.2. Data Collection

Patient characteristics and the Lichtiger score were assessed, and serum and stool samples were collected at baseline (time of steroid initiation) and after 4 weeks of corticosteroid therapy. Further follow-up was evaluated with telephone interviews 3 and 12 months after corticosteroid initiation for assessment of steroid dependence and colectomy rate. Patients refractory to corticosteroids could have an unscheduled study visit to start rescue therapy with infliximab or a calcineurin inhibitor and were labeled as nonresponders at week 4.

### 2.3. Primary Endpoint

The primary endpoint was Lichtiger response to systemic corticosteroids after 4 weeks of treatment. Lichtiger response was defined as a decrease in the Lichtiger score of ≥50% from baseline to week 4 [15] (Table 1). The Lichtiger score is determined by eight variables: diarrhea, nocturnal stools, visible blood in stool, fecal incontinence, abdominal pain/cramping, general well-being, abdominal tenderness, and need for antidiarrheals. The score ranges from 0 (no activity) to 21 points (maximal activity) [15,16].

### 2.4. Secondary Endpoints

Secondary endpoints at week 4 are outlined in Table 1. In addition to the Lichtiger score, we assessed a combined endpoint comprising the Lichtiger score and the biomarkers calprotectin and CRP as used in prior publications investigating treatment efficacy in UC [15,16,17,18,19,20,21,22,23].

Steroid dependence after three months (defined as unable to reduce corticosteroids below the equivalent of prednisolone 10 mg per day or as recurrent active disease after stopping steroids [24]) and colectomy rates after 12 months were assessed.

Baseline patient demographics, disease characteristics, medical therapies, and biomarkers associated with response to corticosteroids were examined. The accuracy of predicting response to treatment at baseline with available biomarkers and the Lichtiger score was evaluated.

### 2.5. Serum Samples, Fecal Calprotectin/Lipocalin-2

Serum samples were analyzed separately and decentralized at each study center. Fecal calprotectin and fecal lipocalin-2 (LCN-2) were centralized. For calprotectin and LCN-2, the S100A8/S100A9 heterodimer DuoSet ELISA (R&D systems, Minneapolis, MN, USA) and the human lipocalin-2/NGAL DuoSet ELISA (R&D systems, Minneapolis, MN, USA) were used, respectively, according to the manufacturer’s instructions. Individual dilutions were performed for all samples outside the assays’ standard curves to calculate absolute concentrations.

### 2.6. Statistical Analysis

Patient characteristics were reported as absolute and relative frequencies for categorical data and as median and range for numerical data. Comparisons between groups were carried out using Mann–Whitney U, Kruskal–Wallis, chi-square, or Fisher exact tests as appropriate. A *p*-value of 0.05 or less was considered statistically significant. Logistic regression analysis was performed to identify factors associated with nonresponse to corticosteroids. Variables with a *p*-value of <0.2 in the univariable analysis were included in the multivariable model. The accuracy of prediction of response to corticosteroids was assessed using receiver operating characteristic (ROC) curves. All statistical analyses were conducted using R version 4.2.3 [25].

## 3. Results

### 3.1. Clinical Efficacy of Systemic Corticosteroids in Active UC

A total of 103 patients were included in the main efficacy analysis. Despite clinical improvement to corticosteroids, seven patients received additional biologics between baseline and week 4 (Table S1). These seven patients were excluded in a sensitivity analysis (*n* = 96) (Figure S1). Patient characteristics of both cohorts at baseline are shown in Table S2.

A total of 80 (78%) patients received systemic prednisolone and 23 (22%) received methylprednisolone. The median (range) dosage of prednisolone was 50 (25, 100) mg at baseline and 20 (5, 75) mg at week 4, while the median dosage of methylprednisolone was 40 (32, 80) mg at baseline and 10 (10, 20) mg at week 4. A total of 36 (35%) patients reported side effects during the first 4 weeks of treatment, including sleeping disorders (*n* = 11), mood disturbances (*n* = 9), weight gain (*n* = 7), acne (*n* = 5), weakness (*n* = 4), hyperglycemia (*n* = 3), sweating (*n* = 3), and visual impairment (*n* = 3).

**Figure 1:**
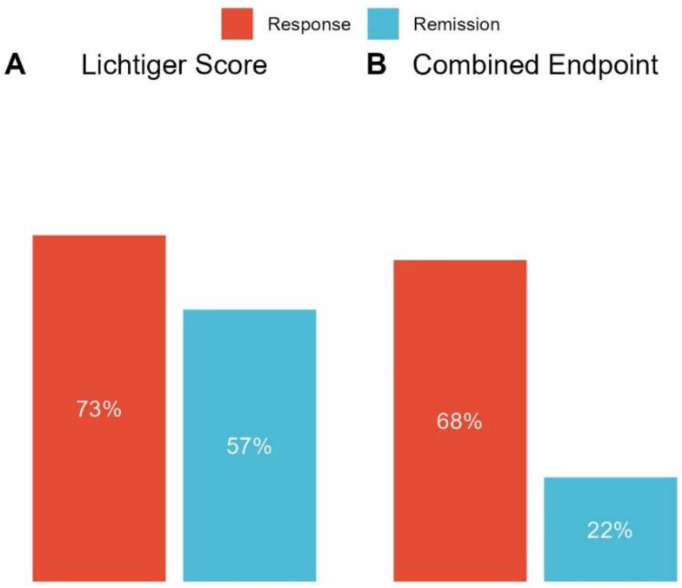
Primary and secondary endpoints. (**A**) Outcome at week 4 after initiation of systemic corticosteroids according to the Lichtiger score (*n* = 103). Clinical response was defined as a decrease in the Lichtiger score of ≥50% from baseline to week 4, while clinical remission was defined as a Lichtiger score ≤ 3 at week 4. (**B**) Outcome at week 4 according to the combined endpoint, including the Lichtiger score, CRP, and calprotectin at week 4 (*n* = 99). Combined response was defined as a decrease in the Lichtiger score of ≥50% and any reduction in CRP and/or calprotectin from baseline to week 4 in patients with elevated biomarkers (CRP and/or calprotectin) at baseline, while combined remission was defined as a Lichtiger score ≤ 3 and CRP < 5 mg/L and/or calprotectin < 250 mg/kg at week 4.

**Figure 2:**
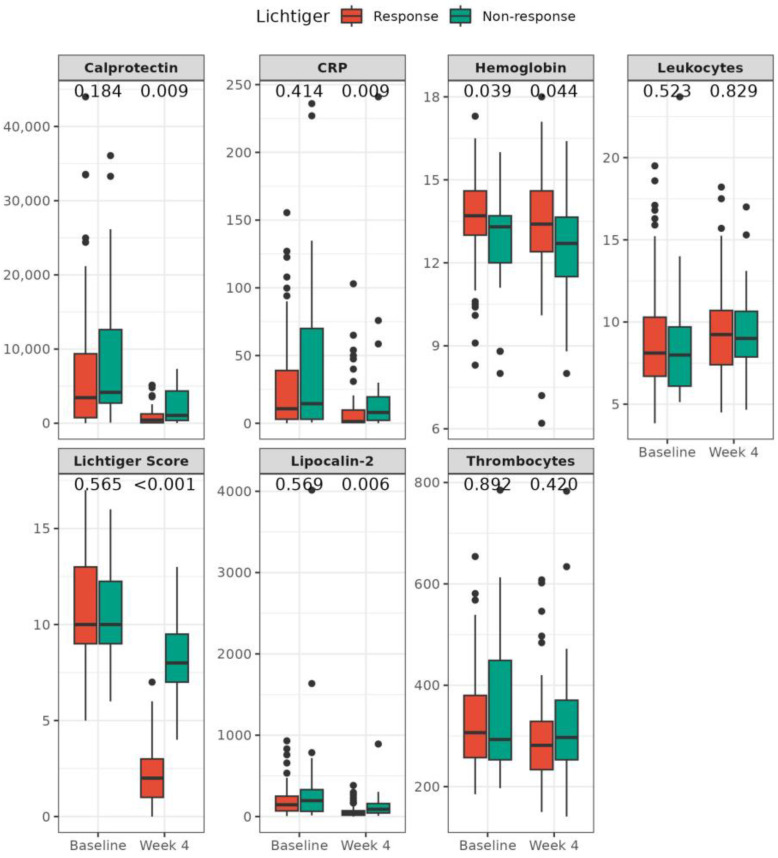
Changes of biomarkers from baseline to week 4. Boxplots for Lichtiger score and biomarkers at baseline and at week 4 (*n* = 103) in patients with Lichtiger response (red) and Lichtiger nonresponse (green). Lichtiger response was defined as a decrease in the Lichtiger score of ≥50% from baseline to week 4. The Mann–Whitney U test was used for comparisons between the groups. *p*-values are provided for comparisons between Lichtiger response and nonresponse groups.

A total of 75 (73%) patients achieved Lichtiger response and 59 (57%) received Lichtiger remission at week 4 (Figure 1A). Patients not achieving Lichtiger response had higher past smoking rates (78% vs. 46%, *p* = 0.005) and lower hemoglobin levels (13.3 (8.0, 16.0) vs. 13.7 (8.3, 17.3), *p* = 0.039) at baseline (Table 2). No differences in fecal or serum inflammation markers and Lichtiger score at baseline were observed. After 4 weeks of treatment, Lichtiger nonresponders had significantly higher fecal calprotectin (1061 (30, 7328) vs. 434 (3, 5129), *p* = 0.009), CRP (8 (0, 241) vs. 1 (0, 103), *p* = 0.009), and fecal LCN-2 (89 (9, 892) vs. 42 (0, 383), *p* = 0.006) levels and lower albumin (4.0 (2.5, 4.6) vs. 4.3 (2.8, 5.1), *p* = 0.005) and hemoglobin (12.7 (8.0, 16.4) vs. 13.4 (6.2, 18.0), *p* = 0.044) levels compared to responders (Figure 2, Table S3).

Fecal calprotectin and CRP levels at baseline and at week 4 correlated with the Lichtiger score at the same time points, while fecal LCN-2 only correlated with the clinical score at week 4 (Figure S2).

### 3.2. Biochemical Efficacy of Systemic Corticosteroids in Active UC

Combined response (*n* = 99) was achieved by 67 (68%) patients and combined remission by 22 (22%) patients (Figure 1B). Patients not achieving combined response had higher rates of biologic experience (44% vs. 21%, *p* = 0.018). 

### 3.3. Steroid Dependence and Colectomy Rates

One out of the 103 patients included in the study was steroid refractory and required rescue therapy with infliximab. The patient was therefore labeled as a nonresponder at week 4. A total of 13 (15%) out of the 87 patients with data at month 3 were steroid dependent. Steroid-dependent patients at month 3 had higher rates of severely active disease (85% vs. 45%, *p* = 0.008) and higher leukocyte levels (10.4 (7.0, 19.5) vs. 8.1 (3.8, 23.7), *p* = 0.006), CRP levels (40 (7, 236) vs. 10 (0, 227), *p* = 0.006), and a higher Lichtiger score (14 (9, 15) vs. 10 (5, 17), *p* = 0.008) at baseline (Table S4). Corticosteroid dose at baseline did not correlate with Lichtiger scores and did not influence response to treatment. One (1%) out of the 70 patients with data at month 12 required colectomy. This patient suffered from severely active colitis at baseline.

### 3.4. Influence of Disease Activity on Response to Corticosteroids

**Figure 3:**
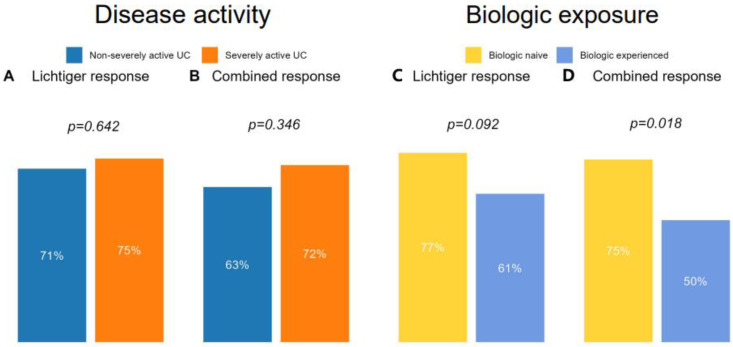
Outcome according to disease activity and biologic exposure. Disease activity: Outcome of patients (*n* = 103) at week 4 after initiation of systemic corticosteroids in relation to disease activity at baseline according to the Lichtiger score. Severely active colitis was defined as a Lichtiger score > 10 at baseline. Severely active colitis: *n* = 48, nonseverely active colitis: *n* = 55. (**A**) Lichtiger response and (**B**) combined response. Biologic exposure: Outcome of patients 4 weeks after initiation of systemic corticosteroids (*n* = 103) according to (**C**) Lichtiger response and (**D**) combined response divided into biologic-naive and biologic-experienced patients. Biologic-experienced patients were defined as patients with prior or ongoing biologic therapy. Biologic-experienced patients: *n* = 28, biologic-naïve patients: *n* = 75. *p*-values between groups are indicated above the graphs.

To estimate the effect of disease activity on the efficacy of corticosteroids, we compared response rates in patients with (Lichtiger score > 10, *n* = 48) and without (Lichtiger score ≤ 10, *n* = 55) severely active colitis (Table S5). Lichtiger response was achieved by 36 (75%) patients with and 39 (71%) patients without severely active colitis at baseline (*p* = 0.642). Equally, no difference could be observed for combined response (Figure 3). Corticosteroid dosing did not differ significantly between groups (Table S5). Although patients with severely active colitis had significantly higher fecal calprotectin, CRP, thrombocyte, and leukocyte levels at baseline and tended to have higher hospitalization rates, levels of all biomarkers and the Lichtiger score aligned between groups after 4 weeks of treatment (Figure S3).

### 3.5. Influence of Medical Therapies on Clinical Response to Corticosteroids

Lichtiger response rates were numerically higher and combined response rates significantly higher (*p* = 0.018) in biologic-naïve patients (*n* = 75) compared to biologic-experienced (*n* = 28) patients (Figure 3). Biologic experience was defined as prior or ongoing biologic therapy. Biologic-experienced patients had longer disease duration (*p* = 0.003) and higher rates of prior corticosteroid (*p* < 0.001) and immunomodulator (*p* = 0.001) exposure (Table S6). The Lichtiger score and biomarkers at baseline were comparable between the two groups, but biologic-naïve patients had a significantly lower Lichtiger score (*p* = 0.015) at week 4 (Figure S4).

Furthermore, we assessed if treatment outcomes differed according to prior corticosteroid and azathioprine exposure. No significant differences could be observed.

### 3.6. Factors Influencing Response to Corticosteroids

Using logistic regression analysis, past smoking history (multivariable: OR 5.38 [1.71, 20.1] for ex-smokers, *p* = 0.003), and baseline hemoglobin levels (multivariable: OR 0.76 [0.57, 0.99] for higher levels, *p* = 0.045) were identified as independent risk factors associated with Lichtiger nonresponse (Table S7). Concerning the combined endpoint, past smoking history (multivariable: OR 2.80 [1.00, 8.58] for ex-smokers, *p* = 0.050), baseline hemoglobin levels (multivariable: OR 0.75 [0.57, 0.97] for higher levels, *p* = 0.029), and biologic experience (multivariable: OR 3.30 [1.08, 10.6] for biologic experience, *p* = 0.036) were independent factors associated with nonresponse to treatment at week 4 (Table S8).

### 3.7. Modelling Response Prediction by Baseline Covariates

**Figure 4:**
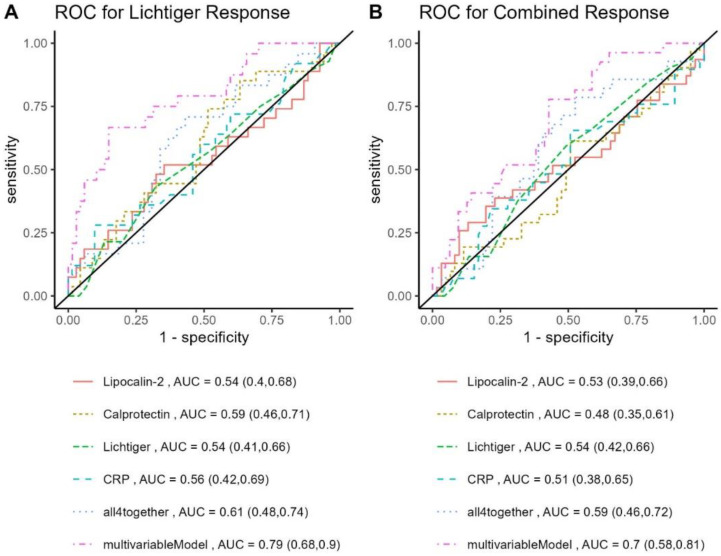
Prediction of response. ROC curves for prediction of response at week 4 before treatment initiation of systemic corticosteroids according to the (**A**) Lichtiger score and (**B**) combined endpoint. Biomarkers (lipocalin-2, calprotectin, and CRP) and the Lichtiger score were analyzed separately and together (all four together). For the multivariable model, all variables with a *p* < 0.2 in the univariable analysis were included.

Concerning the Lichtiger response, the score itself and biomarkers at baseline showed modest ability to predict response to corticosteroids at week 4. Prediction could be improved by combining those parameters (Lichtiger score, CRP, fecal calprotectin, and LCN-2; Lichtiger response: AUC 0.61 [0.48, 0.74]) and using a multivariable model combining factors identified in the univariable analysis with a *p*-value of <0.2 (Lichtiger response: AUC 0.79 [0.68, 0.90]). Combined response could best be predicted by the multivariable model (AUC 0.70 [0.58, 0.81]), including smoking history, use of concomitant immunomodulators, biologic experience, body mass index, and hemoglobin levels at baseline (Figure 4).

### 3.8. Sensitivity Analysis

The sensitivity analysis (*n* = 96) was conducted to exclude potential bias by seven patients initiating biologics concomitantly to corticosteroids between baseline and week 4 despite clinical improvement. No substantial differences to the primary results could be observed.

## 4. Discussion

This prospective, multicenter study highlights the efficacy of systemic corticosteroids in active UC on clinical and biochemical endpoints and suggests that short-term response to treatment is dependent on biologic exposure rather than on baseline disease activity, whereas disease activity impacts longer-term steroid dependence.

Despite the common use of systemic corticosteroids in active UC and the ongoing recommendation of their usage in treatment guidelines [2,3], actual data on the efficacy of this treatment are rare. In the initial studies investigating systemic corticosteroids for the treatment of UC more than 60 years ago, only 24% of patients with mild to moderate colitis and 33% with severe colitis did not achieve clinical remission, but the quality of evidence from these data is limited due to the restricted sample size and the lack of validated clinical scores and biomarkers to measure disease activity [6,7,8]. Furthermore, these data were assessed before the availability of advanced therapies for the treatment of inflammatory bowel diseases. Recent studies investigating the use of corticosteroids in acute severe UC reported treatment failure rates of 26 to 33% [9,10,11]. Another study including patients with newly diagnosed moderate to severe UC found a treatment failure rate of 37% after 3 months [12]. Although we studied a slightly different patient collective including patients with all kinds of baseline disease activity, the rates of clinical nonresponse of around 25% according to the Lichtiger score in our study are in line with published studies.

Interestingly, baseline disease activity did not seem to influence short-term response in our cohort. Even in the subgroup of patients with severely active colitis according to a Lichtiger score > 10, we found high clinical response rates of 75% according to the Lichtiger score. Ben-Horin et al. recently reported similar response rates in patients with acute severe colitis seven days after treatment induction [9]. Of note, the definition of severe colitis (Lichtiger score > 10 vs. Lichtiger score ≥ 10) and Lichtiger response (a ≥50% drop in Lichtiger score vs. a drop of >3 points) were stricter in our cohort.

Rates of steroid dependence after three months (15%) and colectomy rates within 12 months (1%) were low in our cohort. In patients suffering from acute severe colitis, long-term colectomy rates even in responders to intravenous corticosteroids or infliximab were previously reported to be 25% [26]. This difference may be explained by the inclusion of patients with mild and moderate disease activity and by the shorter follow-up in our cohort. Patients with higher disease activity at baseline had an increased risk of becoming steroid dependent and may therefore profit from an early introduction of advanced therapies in clinical practice to avoid relevant side effects of long-term corticosteroid therapy. As shown in our study, 35% of patients already experienced side effects to corticosteroids within 4 weeks of therapy.

According to our data, biologic exposure seems to be negatively associated with response to corticosteroids. Overlapping cofactors such as longer disease duration and higher rates of pretreatment with corticosteroids and immunomodulators in biologic-exposed patients may bias these findings. However, when analyzed by itself, response rates did not differ regarding pretreatment with either corticosteroids or immunomodulators. The lower treatment efficacy of biologics and small molecules in UC patients who were previously exposed to biologics, especially TNF alpha antibodies, has been observed for the most advanced therapies [27,28,29,30]. We are not aware of this already being reported for corticosteroids. Together, this suggests that biologic-exposed patients represent a subgroup of UC patients that are especially difficult to treat.

Factors associated with Lichtiger and combined nonresponse to corticosteroids were prior smoking and lower baseline hemoglobin levels. The association of lower hemoglobin levels indicating treatment failure to corticosteroids in UC patients has been reported previously [11]. Furthermore, Ardizzone et al. published that female sex was associated with better outcomes three months after initiation of corticosteroids, a finding we could not confirm [12].

Fecal calprotectin has been shown to predict one-year sustained response and mucosal healing at treatment week 8 or after induction therapy of biologics [31,32,33]. In patients with acute severe colitis receiving intravenous corticosteroids, a baseline calprotectin level above 800 µg/g was able to predict the need for rescue therapy with an acceptable sensitivity of 80% but a weak specificity [11]. An ulcerative colitis endoscopic index of severity (UCEIS) > 6 at admission and fecal calprotectin levels >1000 µg/g on day 3 were independent predictors of steroid failure in acute severe colitis according to a prospective, single-center study [34]. We found that a combination of the Lichtiger score and biomarkers (CRP, fecal calprotectin, and fecal LCN-2) was of limited use to predict response, and the use of a multivariable model identified by regression analysis at baseline improved prediction only slightly.

The limitations of our study include the restricted sample size, the lack of data on endoscopic or histological endpoints, and the lack of a placebo group. Not all patients had a follow-up available until month 12, and data on dose adaptations of concomitant therapies are lacking. Another important limitation is the absence of a uniform dosing and tapering scheme of corticosteroids, but this may not have impacted results substantially because starting doses did not differ according to baseline disease activity and did not impact response rates. Further, this may not have affected rates of steroid dependence at month 3 because even longer tapering schemes due to higher baseline doses should lead to doses under 10 mg after 12 weeks if dosing had not been increased again or the reduction was stopped.

The strength of our study comprises the prospective, multicenter design with real-world data from a cohort with active UC with various disease activities. Further, the study provides high-quality evidence for corticosteroid usage in the biologic era and suggests for the first time the negative association of biologic exposure with clinical and biochemical response to corticosteroids.

In conclusion, our study confirms a good short-term efficacy of systemic corticosteroids on symptom relief and biomarker endpoints in UC patients independent of disease activity and indicates a negative influence of biologic exposure on response rates. Patients with a high baseline disease activity had a higher risk of steroid dependence, supporting early concomitant introduction of immunomodulators or advanced therapies in this subset of patients.

## Figures and Tables

**Table 1:** Endpoints. Definition of the primary and secondary endpoints at week 4 used in the study.

Endpoint	Definition
	Primary endpoint
Lichtiger response	Decrease in the Lichtiger score of ≥50% from baseline to week 4
	Secondary endpoints
Lichtiger remission	Lichtiger score ≤ 3 after 4 weeks
Combined response	Decrease in the Lichtiger score of ≥50% from baseline to week 4 and any reduction of CRP and/or calprotectin in patients with elevated CRP or calprotectin at baseline
Combined remission	Lichtiger score ≤ 3 and CRP < 5 mg/L and/or calprotectin < 250 mg/kg after 4 weeks in patients with elevated CRP and/or calprotectin at baseline

**Table 2:** Patient characteristics. Characteristics of patients (*n* = 103) at baseline according to Lichtiger response at week 4. Lichtiger response was defined as a decrease in the Lichtiger score of ≥50% from baseline to week 4. Data are shown as median (range) or *n* (%). *p*-values were calculated with chi-square or Mann–Whitney-U tests as appropriate.

	Response (*n* = 75)	Nonresponse (*n* = 28)	*p*-Value
Age (years)	38 (20, 75)	40 (21, 82)	0.412
Female sex	33 (45)	12 (43)	0.875
Body mass index	23.6 (16.6, 34.8)	24.5 (19.7, 36.8)	0.126
Disease duration at study inclusion (years)	3 (0, 59)	6 (0, 34)	0.466
Disease extent/Montreal classification			0.344
E1	8 (11)	3 (11)	
E2	34 (45)	8 (30)	
E3	33 (44)	16 (59)	
Unknown		1	
Severely active colitis	36 (48)	12 (43)	0.642
Hospitalization	29 (41)	12 (43)	0.855
Active smoking	5 (7)	4 (14)	0.266
Past smoking	33 (46)	21 (78)	0.005
Prednisolone dose (mg)	50 (25, 75)	50 (25, 100)	0.416
Methylprednisolone dose (mg)	40 (32, 64)	64 (48, 80)	0.013
Concomitant biologics	11 (15)	7 (25)	0.249
Concomitant immunomodulators	4 (5)	3 (11)	0.386
Concomitant oral 5-ASA	56 (75)	20 (71)	0.740
Prior use of corticosteroids	32 (43)	13 (46)	0.732
Hemoglobin (g/dL)	13.7 (8.3, 17.3)	13.3 (8.0, 16.0)	0.039
Leukocytes (10^9^/L)	8.1 (3.8, 19.5)	8.0 (5.1, 23.7)	0.525
Thrombocytes (10^9^/L)	306 (185, 654)	293 (197, 785)	0.896
C-reactive protein (mg/L)	11 (0, 156)	14 (1, 236)	0.417
Albumin (g/dL)	4.1 (1.6, 5.3)	3.9 (2.5, 5.0)	0.450
Calprotectin (mg/kg)	3452 (8, 43998)	4166 (84, 36076)	0.185
LCN-2 (ng/mL)	145 (6, 931)	196 (14, 4014)	0.572
Lichtiger score	10 (5, 17)	10 (6, 16)	0.568

## Data Availability

The datasets used and analyzed during the current study are available from the corresponding author upon reasonable request.

## Supplementary Materials

The following are available online at https://www.mdpi.com/article/10.3390/jcm12144853/s1, Table S1: Excluded patients, Figure S1: Flow chart, Table S2: Baseline characteristics, Table S3: Characteristics at week 4, Figure S2: Heatmap of Spearman correlations, Table S4: Characteristics steroid dependence, Table S5: Characteristics disease severity, Figure S3: Boxplots disease severity, Table S6: Characteristics biologic exposure, Figure S4: Boxplots biologic exposure, Table S7: Logistic regression analysis for Lichtiger non-response, Table S8: Logistic regression analysis for combined non-response.
