# Supplementary material for: Factors Associated with Response to Systemic Corticosteroids in Active Ulcerative Colitis: Results from a Prospective, Multicenter Trial
Source: J Clin Med. 2023 Jul 24;12(14):4853. doi: 10.3390/jcm12144853 (PMC10382050; doi:10.3390/jcm12144853)
Supplement: Supplementary file 1 [file jcm-12-04853-s001.zip › jcm-2482132-supplementary.pdf]

Supp. Table S1. Excluded patients. Seven patients with clinical improvement to corticosteroids received additional biologics between baseline and week 4. Displayed are types of drugs and reasons for initiation of these drugs in the individual patients. At week 4, these patients were classified according to their actual biochemical and clinical score values.

Patient	Drug	Reasons for initation	Lichtiger Response at week 4
Patient 8	Infliximab	Initiation of biologic despite clinical improvement to corticosteroids	Non-response
Patient 12	Infliximab	Response to corticosteroids, planned initiation of additional biologic due to high disease activity at baseline	Response
Patient 24	Infliximab	Initiation of biologic despite clinical improvement to corticosteroids	Non-response
Patient 26	Infliximab	Initiation of biologic despite clinical improvement to corticosteroids	Non-response
Patient 77	Golimumab	Clinical improvement to corticosteroids, planned initiation of additional biologic due to high disease activity at baseline	Non-response
Patient 85	Infliximab	Response to corticosteroids, planned initiation of additional biologic due to high disease activity at baseline	Response
Patient 91	Infliximab	Response to corticosteroids, additional initiation of biologic due to extraintestinal manifestations (arthralgia)	Response

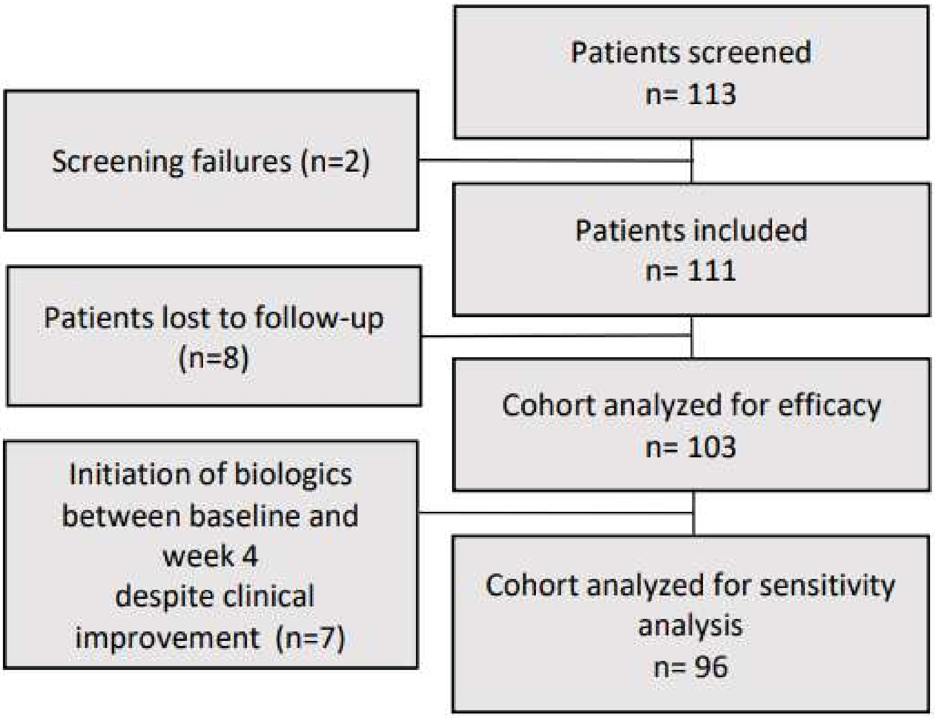

Supp. Figure S1. Flow chart of recruited and subsequently excluded and included study subjects. 113 patients were screened for the study, 2 patients were screening failures and 8 patients were lost to follow-up. Therefore, 103 patients were included in the main efficacy analysis. 7 patients with clinical improvement to corticosteroids received additional biologics between baseline and week 4 and were consequently excluded in the sensitivity analysis.

Supp. Table S2. Characteristics of included patients in the main efficacy analysis (n=103) and the sensitivity analysis (n=96) at baseline. Data are shown as median (range) or n (%).

	Main cohort (n=103)	Sensitivity analysis cohort (n=96)
Age (years)	38 (20, 82)	39 (20, 82)
Female sex	45 (44)	45 (47)
Body mass index	23.8 (16.6, 36.8)	23.8 (16.6, 34.8)
Disease duration at study inclusion (years)	4 (0, 59)	4 (0, 59)
Disease extent/Montreal classification		
E1	11 (11)	10 (11)
E2	42 (41)	40 (42)
E3	49 (48)	45 (47)
Unknown	1	1
Severely active colitis	48 (47)	43 (45)
Hospitalisation	41 (41)	35 (38)
Active smoking	9 (9)	8 (9)
Past smoking	54 (55)	50 (54)
Concomitant biologics	18 (17)	18 (19)
Concomitant immunomodulators	7 (7)	7 (7)
Concomitant oral 5-ASA	76 (74)	71 (74)
Prior use of corticosteroids steroids	45 (44)	43 (45)
Hemoglobin (g/dL)	13.5 (8.0, 17.3)	13.6 (8.3, 17.3)
Leukocytes (10^9/L)	8.0 (3.8, 23.7)	7.9 (3.8, 23.7)
Thrombocytes (10^9/L)	303 (185, 785)	302 (185, 785)
C-reactive protein (mg/L)	13 (0, 236)	11 (0, 236)
Albumin (g/dL)	4.1 (1.6, 5.23)	4.1 (1.6, 5.3)
Calprotectin (mg/kg)	3905 (8, 43998)	3516 (8, 43998)
LCN-2 (ng/ml)	147 (6, 4014)	143 (6, 1636)
Lichtiger score	10 (5, 17)	10 (5, 17)

Montreal classification: E1 = proctitis, E2 = left-sided colitis, E3 = pancolitis, LCN-2 = Lipocalin-2

Supp. Table S3. Characteristics of patients (n=103) at week 4 divided into patients with response and non-response according to Lichtiger score at week 4. Lichtiger response was defined as decrease of Lichtiger score ≥ 50% from baseline to week 4. Data are shown as median (range). P-values were calculated with Chi-square or Mann Whitney U test as appropriate.

	Response (n=75)	Non-response (n=28)	p-value
Body mass index	24.0 (17.6, 35.5)	23.5 (18.8, 36.4)	0.996
Hemoglobin (g/dL)	13.4 (6.2, 18.0)	12.7 (8.0, 16.4)	0.044
Leukocytes (109/L)	9.2 (4.5, 18.2)	9.0 (4.7, 17.0)	0.832
Thrombocytes (109/L)	282 (150, 608)	297 (141, 783)	0.423
C-reactive protein (mg/L)	1 (0, 103)	8 (0, 241)	0.009
Albumin (g/dL)	4.3 (2.8, 5.1)	4.0 (2.5, 4.6)	0.005
Calprotectin (mg/kg)	434 (3, 5129)	1061 (30, 7328)	0.009
LCN-2 (ng/ml)	42 (0, 383)	89 (9, 892)	0.006
Lichtiger score	2 (0, 7)	8 (4, 13)	<0.001

LCN-2 = Lipocalin-2

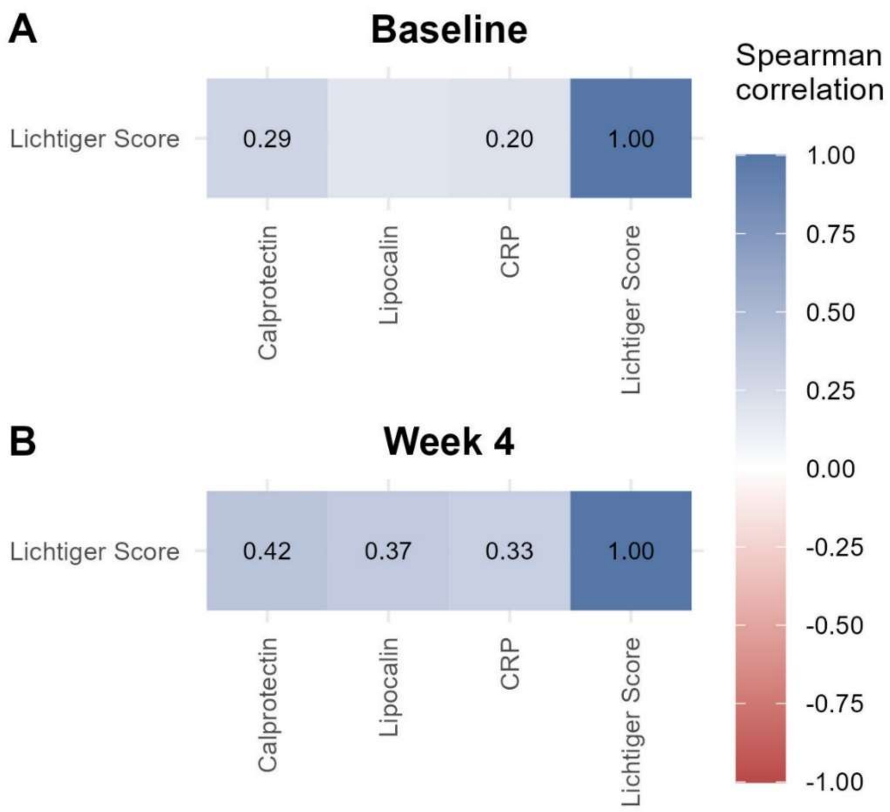

Supp. Figure S2. Heatmap of Spearman correlations between Lichtiger Score and biomarkers at baseline **(A)** and at week 4 **(B)**. All indicated values have a p-value <0.05.

Supp. Table S4. Baseline characteristics of patients (n=87) divided into patients with steroid dependence and without steroid dependence at month 3. Steroid dependence was defined as patients unable to reduce corticosteroids below the equivalent of prednisolone 10 mg/day or as recurrent active disease after stopping steroids. Data are shown as median (range) or n (%). P-values were calculated with Chi-square or Mann Whitney U test as appropriate.

	No steroid dependance (n=74)	Steroid dependance (n=13)	p-value
Age (years)	42 (20, 75)	33 (22, 54)	0.120
Female sex	35 (47)	5 (38)	0.556
Body mass index	24.1 (17.2, 34.8)	22.8 (16.9, 36.8)	0.508
Disease duration at study inclusion (years)	4 (0, 59)	3 (0, 32)	0.692
Disease extent/Montreal classification			0.908
E1	8 (11)	1 (8)	
E2	29 (39)	6 (46)	
E3	37 (50)	6 (46)	
Severely active colitis	33 (45)	11 (85)	0.008
Hospitalisation	28 (39)	8 (62)	0.139
Active Smoking	5 (7)	2 (15)	0.295
Past smoking	37 (52)	6 (50)	0.892
Prednisolone dose (mg)	50 (25, 100)	50 (50, 75)	0.477
Methyprednisolone dose (mg)	40 (32, 64)	72 (64, 80)	0.032
Concomitant biologics	12 (16)	3 (23)	0.690
Concomitant immunomodulators	5 (7)	0 (0)	>0.999
Concomitant oral 5-ASA	54 (73)	9 (69)	0.747
Prior use of corticosteroids	31 (42)	5 (38)	0.817
Hemoglobin (g/dL)	13.5 (8.0, 17.3)	13.6 (10.4, 16.2)	0.808
Leukocytes (10^9/L)	8.1 (3.8, 23.7)	10.4 (7.0, 19.5)	0.006
Thrombocytes (10^9/L)	302 (185, 654)	350 (227, 568)	0.218
C-reactive protein (mg/L)	10 (0, 227)	40 (7, 236)	0.006
Albumin (g/dL)	4.1 (2.1, 5.3)	3.9 (1.6, 4.8)	0.535
Calprotectin (mg/kg)	3516 (46, 33286)	8725 (1921, 43998)	0.062
LCN-2 (ng/ml)	136 (6, 719)	196 (62, 4014)	0.059
Lichtiger score	10 (5, 17)	14 (9, 15)	0.008

Montreal classification: E1 = proctitis, E2 = left-sided colitis, E3 = pancolitis, LCN-2 = Lipocalin-2

Supp. Table S5. Baseline characteristics of patients with and without severely active colitis according to Lichtiger score at baseline (n=103). Severely active colitis was defined as Lichtiger score > 10 at baseline. Data are shown as median (range) or n (%). P-values were calculated with Chi-square or Mann-Whitney U test as appropriate.

	Non-severely active colitis (n=55)	Severely active colitis (n=48)	p-value
Age (years)	38 (20, 82)	40 (21, 75)	0.854
Female sex	21 (39)	24 (50)	0.259
Body mass index	24.3 (16.6, 34.2)	23.4 (16.9, 36.8)	0.559
Disease duration at study inclusion (years)	4 (0, 37)	4 (0, 59)	0.399
Disease extent/Montreal classification			0.658
E1	6 (11)	5 (10)	
E2	20 (37)	22 (46)	
E3	28 (52)	21 (44)	
Unknown	1		
Hospitalisation	17 (33)	24 (51)	0.064
Active smoking	4 (8)	5 (11)	0.732
Past smoking	31 (60)	23 (50)	0.340
Prednisolone dose (mg)	50 (25, 100)	50 (25, 75)	0.075
Methyprednisolone dose (mg)	40 (32, 64)	48 (40, 80)	0.052
Concomitant biologics	7 (13)	11 (23)	0.174
Concomitant immunomodulators	3 (6)	4 (8)	0.702
Concomitant oral 5-ASA	41 (75)	35 (73)	0.851
Prior use of corticosteroids	22 (40)	23 (48)	0.419
Hemoglobin (g/dL)	13.7 (8.8, 17.3)	13.3 (8.0, 16.2)	0.118
Leukocytes (10^9/L)	7.1 (4.5, 18.6)	8.9 (3.8, 23.7)	0.006
Thrombocytes (10^9/L)	287 (185, 785)	318 (219, 613)	0.035
C-reactive protein (mg/L)	8 (1, 156)	25 (0, 236)	0.041
Albumin (g/dL)	4.1 (3.0, 5.3)	3.9 (1.6, 5.0)	0.055
Calprotectin (mg/kg)	2999 (8, 33550)	6029 (46, 43998)	0.007
LCN-2 (ng/ml)	121 (9, 1636)	169 (6, 4014)	0.080
Lichtiger score	9 (5, 10)	13 (11, 17)	<0.001

Montreal classification: E1 = proctitis, E2 = left-sided colitis, E3 = pancolitis, LCN-2 = Lipocalin-2

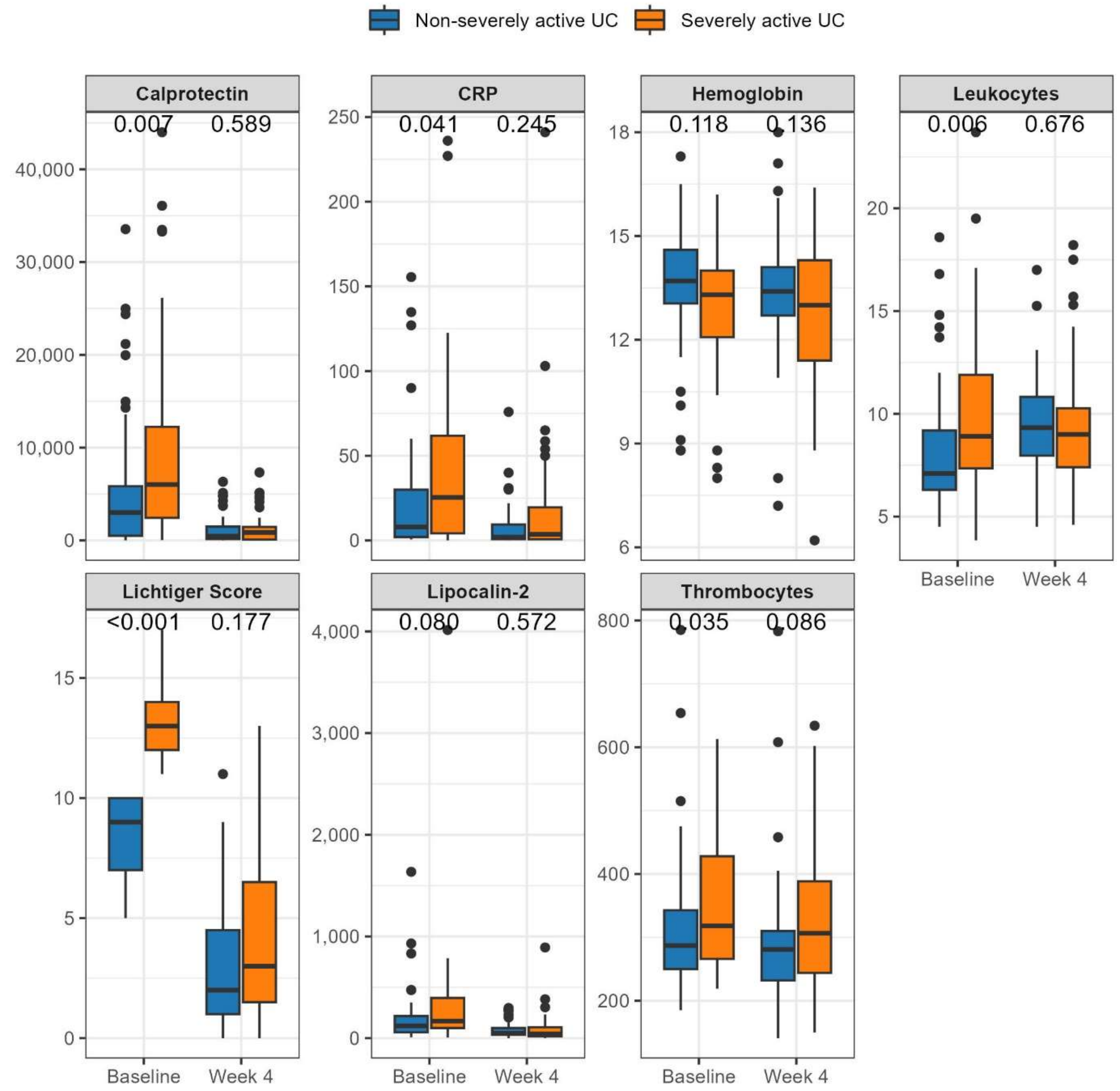

Supp. Figure S3. Boxplots for changes of Lichtiger Score and biochemical parameter from baseline to week 4 (n=103) divided into patients with and without severely active according to Lichtiger Score at baseline. Severely active colitis was defined as Lichtiger score > 10 at baseline. The Mann Whitney U test was used for comparisons between the groups and according p-values are provided.

Supp. Table S6. Baseline characteristics concerning biologic experienced and biologic naïve patients (n=103). Biologic experienced patients were defined as patients with prior or ongoing biologic therapy. Data are shown as median (range) or n (%). P-values were calculated with Chi-square or Mann-Whitney U test as appropriate.

	Biologic experienced (n=28)	Bio-naive (n=75)	p-value
Age (years)	38 (31, 55)	41 (31, 58)	0.750
Female sex	17 (61)	28 (38)	0.038
Body mass index	23.3 (20.7, 27.5)	23.9 (21.9, 26.2)	0.646
Disease duration at study inclusion (years)	9 (5, 16)	2 (0, 10)	0.003
Disease extent/Montreal classification			0.410
E1	1 (4)	10 (14)	
E2	13 (46)	29 (39)	
E3	14 (50)	35 (47)	
Unknown	1		
Hospitalisation	12 (46)	29 (40)	0.568
Active smoking	4 (15)	5 (6.9)	0.251
Past smoking	14 (54)	40 (56)	0.881
Prednisolone dose (mg)	50 (50, 50)	50 (50, 50)	0.216
Methylprednisolone dose (mg)	40 (40, 54)	48 (44, 64)	0.156
Concomitant biologics	18 (64)	0 (0)	<0.001
Concomitant immunomodulators	2 (7)	5 (7)	>0.999
Concomitant oral 5-ASA	23 (82)	53 (71)	0.239
Prior use of coticosteroids	22 (79)	23 (31)	<0.001
Hemoglobin (g/dL)	13.4 (11.8, 14.5)	13.6 (13.1, 14.6)	0.377
Leukocytes (10^9/L)	7.8 (6.5, 8.8)	8.6 (6.8, 10.2)	0.341
Thrombocytes (10^9/L)	299 (262, 358)	303 (253, 400)	0.864
C-reactive protein (mg/L)	8 (2, 20)	16 (3, 43)	0.160
Albumin (g/dL)	4.1 (3.5, 4.5)	4.1 (3.8, 4.4)	0.933
Calprotectin (mg/kg)	2899 (1446, 10458)	4,310 (1870, 9998)	0.578
LCN-2 (ng/ml)	132 (69, 258)	164 (68, 275)	0.970
Lichtiger score	11 (9, 13)	10 (9, 13)	0.652

Montreal classification: E1 = proctitis, E2 = left-sided colitis, E3 = pancolitis, LCN-2 = Lipocalin-2

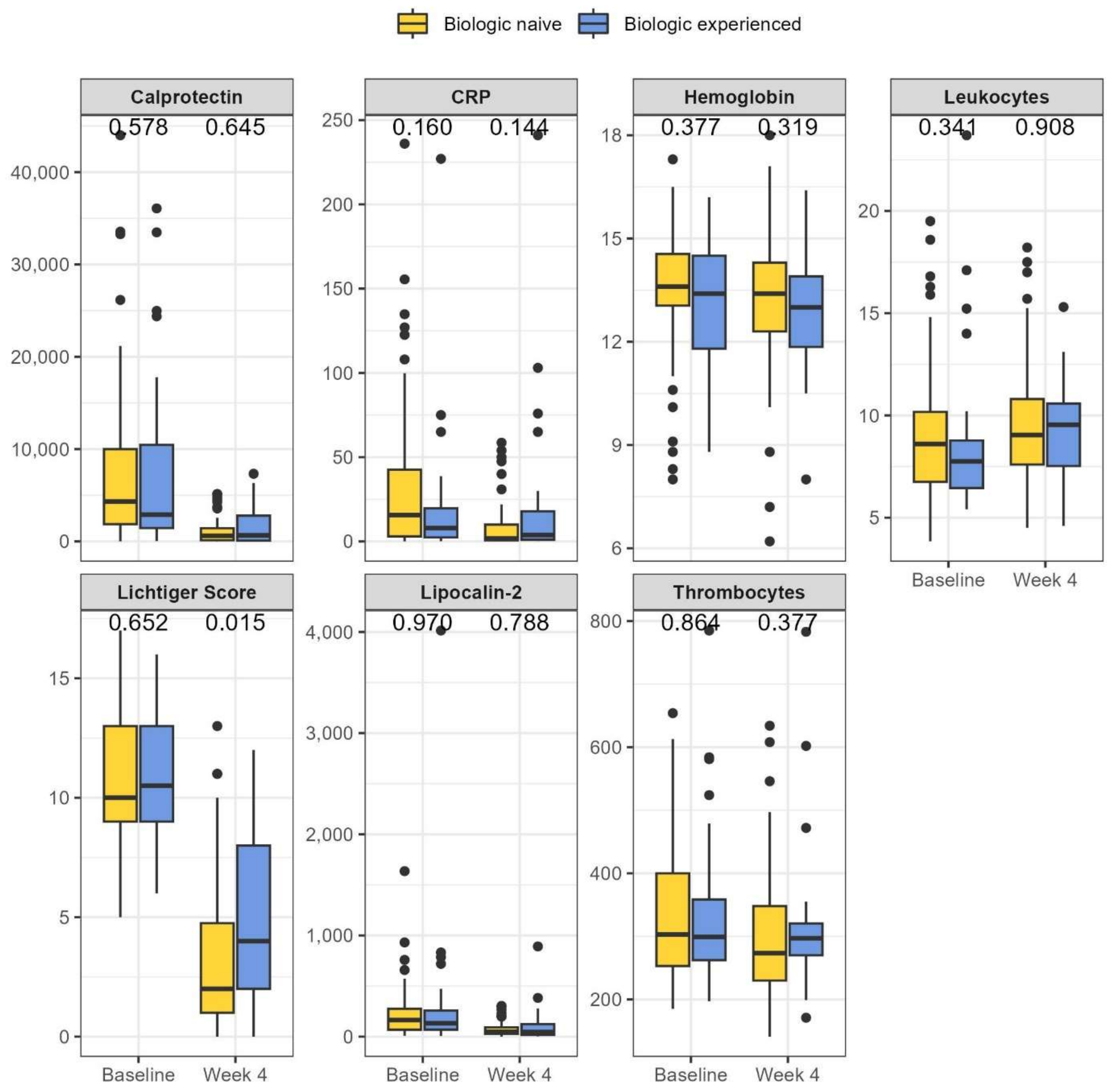

Supp. Figure S4. Boxplots for changes of Lichtiger Score and biomarkers from baseline to week 4 (n=103) divided into biologic naïve (n=28) and biologic experienced (n=75) patients. Biologic experienced patients were defined as patients with prior or ongoing biologic therapy. The Mann-Whitney-U test was used for comparisons between the groups and according p-values are provided.

Supp. Table S7. Logistic regression analysis for Lichtiger non-response. The multivariable analysis includes variables of the univariable analysis with a p-value < 0.2. CRP, calprotectin, LCN-2 and thrombocytes were analyzed with logarithmic scores.

		univariable			multivariable			
Characteristic	N	OR[1]	95% CI[1]	p-value	OR[1]	95% CI[1]	p-value	VIF[1]
Age (years)	102	1.01	0.98, 1.04	0.475				
Sex	102			0.875				
m		—	—					
w		0.93	0.38, 2.23					
Smoking in the past	98			0.004			0.003	1.1
No		—	—		—	—		
Yes		4.03	1.53, 12.1		5.38	1.71, 20.1		
Active Smoking	99			0.277				
No		—	—					
Yes		2.20	0.51, 8.99					
Hospitalisation	99			0.855				
No		—	—					
Yes		1.09	0.44, 2.63					
Years from diagnosis to study inclusion	101	1.01	0.97, 1.05	0.671				
Disease extention	102			0.333				
Left-sided colitis		—	—					
Pancolitis		2.06	0.79, 5.69					
Proctitis		1.59	0.30, 7.06					
Concomitant immunomodulators	103			0.355				
No		—	—					
Yes		2.13	0.40, 10.3					
Prior use of corticosteroids	103			0.732				
No		—	—					
Yes		1.16	0.48, 2.79					
Biologics experienced	103			0.099			0.091	1.1
No		—	—		—	—		
Yes		2.21	0.86, 5.61		2.81	0.85, 9.71		
BMI	98	1.11	0.99, 1.24	0.069	1.13	0.98, 1.31	0.087	1.1
Hemoglobin	97	0.76	0.59, 0.97	0.026	0.76	0.57, 0.99	0.045	1.1
Leukocytes	97	0.96	0.83, 1.09	0.570				
Thrombocytes	95	1.53	0.34, 6.59	0.572				
CRP	97	1.14	0.87, 1.52	0.338				
Albumin	74	0.83	0.39, 1.82	0.640				
Calprotectin	95	1.25	0.93, 1.74	0.139	1.07	0.73, 1.61	0.720	1.2
Lipocalin-2	95	1.18	0.81, 1.77	0.389				
Severely active colitis	103			0.641				
no		—	—					
yes		0.81	0.33, 1.94					

[1]OR = odds Ratio, CI = confidence interval, VIF = variance inflation factor, CRP = C-reactive protein, BMI = body mass index

Supp. Table S8. Logistic regression analysis for combined non-response. The multivariable analysis includes variables of the univariable analysis with a p-value < 0.2. CRP, calprotectin, LCN-2 and thrombocytes were analyzed with logarithmic scores.

		univariable			multivariable			
Characteristic	N	OR[1]	95% CI[1]	p-value	OR[1]	95% CI[1]	p-value	VIF[1]
Age (years)	98	1.01	0.98, 1.04	0.501				
Sex	98			0.651				
m		—	—					
w		0.82	0.34, 1.92					
Smoking in the past	95			0.075			0.050	1.0
No		—	—		—	—		
Yes		2.26	0.92, 5.89		2.80	1.00, 8.58		
Active Smoking	96			0.424				
No		—	—					
Yes		1.78	0.41, 7.23					
Hospitalisation	96			0.971				
No		—	—					
Yes		1.02	0.42, 2.41					
Years from diagnosis to study inclusion	97	1.00	0.96, 1.04	0.937				
Disease extention	98			0.407				
Left-sided colitis		—	—					
Pancolitis		1.86	0.75, 4.81					
Proctitis		1.55	0.29, 7.11					
Concomitant immunomodulators	99			0.161			0.128	1.1
No		—	—		—	—		
Yes		3.05	0.63, 16.3		4.36	0.64, 30.5		
Prior use of corticosteroids	99			0.634				
No		—	—					
Yes		1.23	0.52, 2.88					
Biologics experienced	99			0.020			0.036	1.1
No		—	—		—	—		
Yes		2.94	1.18, 7.44		3.30	1.08, 10.6		
BMI	94	1.11	1.00, 1.24	0.054	1.12	0.99, 1.28	0.078	1.0
Hemoglobin	94	0.79	0.62, 1.00	0.052	0.75	0.57, 0.97	0.029	1.1
Leukocytes	94	0.92	0.80, 1.05	0.235				
Thrombocytes	92	0.89	0.20, 3.69	0.872				
CRP	94	1.00	0.77, 1.30	>0.999				
Albumin	71	1.18	0.57, 2.62	0.664				
Calprotectin	92	1.06	0.81, 1.41	0.684				
Lipocalin-2	92	0.96	0.65, 1.41	0.823				
Severely active colitis	99			0.344				
no		—	—					
yes		0.66	0.28, 1.55					

[1]OR = odds ratio, CI = confidence interval, VIF = variance inflation factor, CRP = C-reactive protein, BMI = body mass index
